# Assessment of Phenol and Antioxidant Content of Olive Varieties and Their Potential Health Benefits for Colon Health

**DOI:** 10.1155/2023/9165902

**Published:** 2023-10-12

**Authors:** Baraa Jarwan, Jawad Tawalbeh, Ruba Malkawi

**Affiliations:** ^1^School of Pharmacy, Jadara University, P.O. Box 733, Irbid 21110, Jordan; ^2^School of Business, Teesside University, Campus Heart, Southfield Rd, Middlesbrough TS1 3BX, Middlesbrough, UK

## Abstract

In this study, four different olive fruit and leaf varieties collected in Jordan were assessed for quality using both chemical and biological methods. To quantify the phenol and antioxidant content in the olive fruit and leaf extracts, a validated UV method was employed. The antioxidant activity and total phenolic content of fruit and leaf extracts of the olive varieties were measured using the DPPH radical scavenging assay and Folin–Ciocalteu colorimetric method, respectively. The researchers also conducted a biological assay against colon cells to examine the potential health benefits of the olive extracts. The results showed that the phenol content of the samples varied depending on the region they were collected from and that they contained a significant amount of antioxidants. Additionally, it was observed that the samples with higher antioxidant content had lower cell viability against colon cells. Overall, this study suggests that olive extracts may have potential health benefits for colon health and that the phenol and antioxidant content of the extracts can vary depending on the source of the olives.

## 1. Introduction

The olive is the fruit of the evergreen olive tree (*Olea europaea*, Oleaceae), which grows in the Mediterranean region's temperate climate. For thousands of years, olive trees have been reliable food sources [1]. The spread of the olive tree is through the Mediterranean region, thus causing the ease of propagation by seed in the fruit transportable branches or “ovules” (big lumps on tree trunks that produce rooted shoots). Every country in the Mediterranean has its own distinctive cultivars, with some orchards dating back hundreds of years [2].

One of the most vital elements of the Mediterranean diet is table olives, a typical Greek product. They have recognized sources of phenolic chemicals with crucial biological characteristics [3]. In addition to the monounsaturated fat, table olives' nutritional advantages relate to minor components such as phenolic chemicals [4]. The quality and quantity of the phenolic chemicals in the phenolic fraction of table olives might vary according to the processing techniques, cultivars, irrigation methods, and drupe maturation [5]. The decrease in oleuropein during the development of the olive fruit and the rise in tyrosol and hydroxytyrosol concentration are the two factors that have the greatest effects on the phenolic fraction. Tyrosol, hydroxytyrosol, and oleanolic acid are the three main phenolic compounds found in table olives [6]. The degree of maturation and method [7] of treatment are the two factors that affect the concertation of the phenolic compounds until they become able to eat [8, 9].

Numerous diseases, including atherosclerosis, cancer, and tissue damage in rheumatoid arthritis, have been linked to oxidative stress [10]. Free radical overproduction in the body can result in oxidative damage to biomolecules such as lipids, proteins, and DNA, which ultimately results in the development of several chronic diseases [11]. Additionally, one significant factor in the deterioration of food products during processing and storage is the lipid peroxidation process [12]. In this instance, lipid peroxidation can be slowed down by the use of antioxidants in food products, especially in lipids and foods containing lipids, to extend the shelf life [12]. Since the carcinogenicity of synthetic antioxidants like butylated hydroxyanisole (BHA) and butylated hydroxytoluene (BHT) has been doubted, their use has been limited in food industries. Subsequently, the search for natural antioxidants, especially from plant sources, has significantly increased recently [13]. Free radical scavenging compounds, such as phenolic compounds (e.g. phenolic acids, flavonoids, quinones, coumarins, lignans, stilbenes, and tannins), nitrogen compounds (alkaloids, amines, and betalains), vitamins, and terpenoids (including carotenoids), are broadly observed in plant species and work as potential antioxidant agents [11]. Plant species that are abundant in antioxidant materials, particularly phenolics, are advised because of their protective effects against cardiovascular diseases, certain malignancies, and many chronic diseases [14].

Due to their phenolic levels, olive oil, leaves, and fruits are regarded as possible natural antioxidant sources [15]. Oleuropein, hydroxytyrosol, tyrosol, and oleanolic acid are phenolic chemicals, flavonoids, and secoiridoids that are found in the leaves and fruits of *O. europaea* trees [16]. Among these substances, the bitter secoiridoid oleuropein is the primary component of the leaf, as well as the seed, pulp, and peel of unripe olives [14, 17]. Oleuropein and its derivatives may have substantial biological effects, including anti-inflammatory, antiatherogenic, antihypertensive, hypoglycemic, hypocholesterolemic, antiproliferative, and antioxidant properties, according to studies [18]. By avoiding LDL oxidation, cancer, and osteoporosis, oleuropein and hydroxytyrosol are beneficial in the treatment of several illnesses, including coronary artery disease. Oleuropein has been shown to be efficient against bacteria, yeasts, fungi, molds, viruses, retroviruses, and other parasites [14]. There are roughly 2500 known kinds of olives, and the International Olive Oil Council classifies 250 of these as commercial cultivars [19].

This study used UV spectroscopy to assess the total phenolic compounds found in olive tree leaves and fruits from four different cultivars of *Olea europaea* L. growing in the north and south of Jordan. The Folin–Ciocalteu technique was used to calculate the total phenolic content. Finally, DPPH techniques were used to evaluate the antioxidant activity of these samples.

## 2. Materials and Methods

### 2.1. Reagents and Chemicals

All solvents used were of HPLC grade. Folin–Ciocalteu reagent and gallic acid 2,2- were purchased from Sigma-Aldrich Chemical, while diphenyl-1-picrylhydrazyl (DPPH•) was obtained from POCH (Poland).

### 2.2. Plant Material and Extraction

The leaves and fruits from four different cultivars (Irbid, Azraq, Mafraq, and Ajloun) were collected, with 600 g of each sample. The collected samples were defatted using hexane and stored in a dark cool place for 24 hours. Subsequently, the extract was decanted and allowed to dry at room temperature (22–25°C).

Next, the defatted leaves and fruits were separately subjected to the soaking method using a mixture of MeOH (methanol) and H_2_O (water) in an 80 : 20 ratio for a duration of two days. In this process, the solvent was added to each cultivar and left to stand for 48 hours. Following this, the samples were sonicated for 10 minutes, and the tubes were then subjected to centrifugation. The upper layer containing the extract was collected for further analysis of total phenol content and antioxidant activity.

### 2.3. Total Phenolic Contents

#### 2.3.1. Preparation of Gallic Acid UV Curve

**Figure 1:**
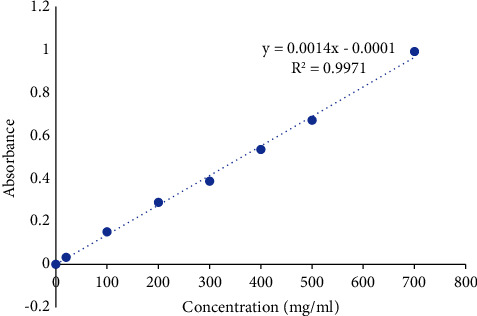
Standard curve of gallic acid.

A typical gallic acid curve was created by preparing the dilutions of 100, 200, 300, 400, 500, 600, and 700 mg/ml in methanol from a standard 1 solution of gallic acid. 150 *µ*l of each of these dilutions was mixed with 450 *µ*l of water, and then 2.5 ml of Folin–Ciocalteu reagent was added and allowed to stand for 5 minutes. Then, 2 ml of 75 g/L sodium carbonate was added. After that, the solution was incubated with shaking for 1.5 hours at 30°C. The absorbance was recorded at 765 nm spectrometrically [20]. The total phenolic content of the olives and leaves was calculated as gallic acid equivalents (mgGAE/g). All the experiments were repeated in triplicate.  Figure 1 displays the standard gallic acid curve and regression equation used to calculate the total phenolic content of the extracts [21].

#### 2.3.2. Calculation of the Total Phenolic Content

Total phenolic contents were measured using the Folin–Ciocalteu method. In brief, 150 *µ*l of each extract was mixed with 2.5 ml of 0.2 N Folin–Ciocalteu reagent stand at room temperature for 5 min. Then, 2 ml sodium bicarbonate solution (75 g/L) was added. The mixture was incubated for 1.5 hours at 30°C. After that, the absorbance was measured at 765 nm using a UV/Vis spectrophotometer (SPUV-26/TECH). Total phenolic contents were measured using a calibration curve gained from measuring the absorbance of seven known concentrations of gallic acid (GA) standard. The concentrations are expressed as milligrams of gallic acid equivalents (mgGAE/g) of dry extract [20].

### 2.4. Scavenging Activity (DPPH) Assay

Using the free radical scavenging technique 2,2-diphenyl-1-picrylhydrazyl (DPPH), the antioxidant properties of the extracts were assessed [22]. The oxidized form of DPPH gives methanol a rich violet colour. An antioxidant compound reduces DPPH by giving it an electron, changing its colour from deep violet to yellow. The absorbance of a freshly made 0.5 mM DPPH solution in 100% methanol was measured at 517 nm. A 2 ml solution of DPPH was added to 150 *µ*l of pure extracts. The samples were measured twice, one at zero time and the second after 30 minutes of standing the solutions in a dark place (all the measurements were conducted at 517 nm). All the samples were measured three times, and the averages for each reading were taken. The following equation was used to calculate the percentage of DPPH that extracts inhibited [22].

(1) $$\begin{array}{c} \%\text{inhibition}=\left(\frac{A-B}{A}\right)*100, \end{array}$$

where *A* is the absorbance of DPPH after the addition of extract at zero time and *B* is the absorbance of a sample taken after 30 minutes of reaction with DPPH.

Then, the %inhibition/100 mg was calculated using the following equation:

(2) $$\begin{array}{c} \frac{\%\text{inhibition}}{100\text{ mg}}=\frac{\left(\text{weight of the extracted sample}*100\right)}{\left(\%\text{inhibition}\right)}. \end{array}$$

### 2.5. Culture of Human Colo205 Cells

Colo205 cells were procured from the American Type Culture Collection (ATCC) located in Manassas, VA. These cells were cultured as monolayers in Roswell Park Memorial Institute (RPMI) 1640 Medium supplied by Life Technologies Limited in Paisley. The medium contained 10% v/v fetal bovine serum (FBS) procured from Atlanta Biologicals located in Flowery Branch, GA, and 1% v/v penicillin-streptomycin (5,000 U/ml) obtained from Life Technologies Corporation in Austin, TX. The cells were incubated in a humidified incubator at 37°C and 5% CO_2_, which was provided by Sanyo Scientific in Wood Dale, IL. The cells were passaged using 0.25% trypsin-EDTA from Life Technologies Corporation in Austin, TX, and the passage number of the cell line was between 2 and 6 [23, 24].

### 2.6. Treating Cells with Olive Fruit and Leaf Extracts

To treat cells with “olive fruit and leaf extracts from different parts of Jordan,” 10,000 cells/well were placed in 96-well tissue microculture plates. After 24 hours of culture, the media was replaced with a media-containing treatment, and it was incubated with the cells for another 24 h [23].

### 2.7. Cell Viability

Cell viability was determined using the MTS cell proliferation assay, CellTiter 96® AQueous One Solution Cell Proliferation Assay (Promega, Madison, WI), and was performed according to the manufacturer's protocol. Cells were treated with “olive fruit and leaf extracts from different parts of Jordan.” Twenty-four hours after treating the cells, 20 *µ*L of MTS assay reagent (3-(4,5-dimethylthiazol-2-yl)-5-(3-carboxymethoxyphenyl)-2-(4-sulfophenyl)-2H-tetrazolium) was added to the cells seeded in the 96-well tissue microculture plate. Cells were then incubated for 4 h in a humidified incubator at 37°C and 5% CO_2_. Then, a SpectraMax Plus 384 microplate reader (Molecular Devices, Sunnyvale, CA) was used to assess the viability of the cells based on the relative level of the soluble formazan formed at *λ*_max_ 490 nm. Untreated Colo 205 cells were used as a control group, and the results were expressed as percent viability relative to the control group [23, 24].

## 3. Results

### 3.1. Total Phenolic Contents

Table 1 displays the total phenolic content of the olive samples, expressed as milligrams of gallic acid equivalents (mgGAE/g) per gram of dry extract. The analysis revealed a total phenolic content ranging from 155.91 ± 0.714 to 413.64 ± 6.81 mg/ml.

Notably, Ajloun leaves exhibited the highest phenolic content compared to the other samples, with Azraq leaves, Maraq fruit, Azraq fruit, Irbid fruit, Mafraq leaves, Ajloun fruit, and Irbid leaves following in that order. It is noteworthy that, with the exception of Azraq and Ajloun, leaves generally displayed a higher total phenolic content than their corresponding fruits. Conversely, in the case of Marfaq and Irbid, fruits typically demonstrated a higher total phenolic content than their respective leaves.

### 3.2. Total Antioxidant Activity

Table 2 presents the range of antioxidant activity, which was found to vary from 473.04 ± 1.96 to 293.49 ± 7.39 mg/ml.

**Figure 2:**
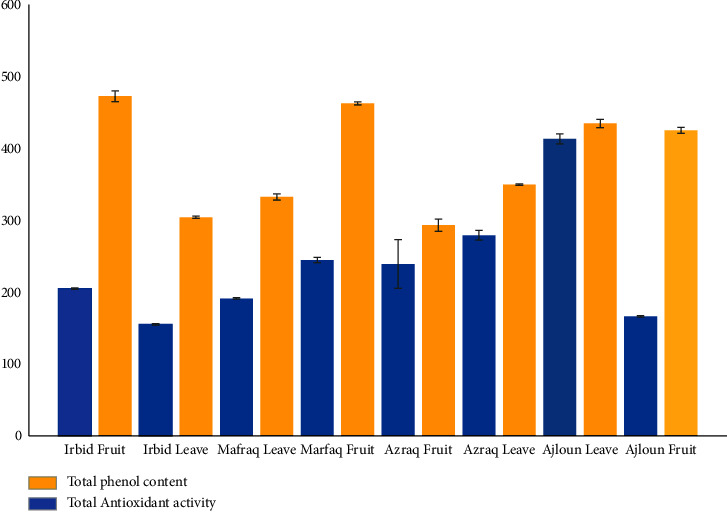
The variations in total phenol content and antioxidant activity of olive fruit and leaf extracts obtained from diverse regions of Jordan.

Notably, the Irbid fruit extract exhibited the highest antioxidant activity, while the Azraq fruit extract had the lowest. Consequently, the Azraq sample was excluded from the biological assay test conducted against stomach cancer cells. Furthermore, Figure 2 provides a comparative analysis of the total phenolic content and antioxidant activity across the various samples of olive fruit and leaves. The figure allows for a direct visual comparison of these two crucial parameters.

### 3.3. Biological Assay

In this study, a biological assay was performed to evaluate the effect of olive fruit and leaf extracts against a stomach cell line.

The results indicated a correlation between apoptosis and antioxidant activity, where higher levels of antioxidant activity led to decreased cell viability.

**Figure 3:**
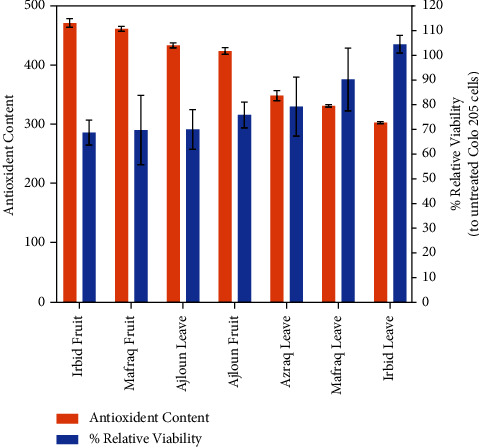
The correlation between the antioxidant activity and the cell viability.

These findings suggest that the olive extracts may have potential as an anticancer agent, as the induction of apoptosis can be a desired outcome in cancer treatment. Further studies are needed to fully understand the mechanism of action and potential therapeutic applications of these extracts. Overall, these results highlight the importance of natural products in the development of novel cancer therapies.  Figure 3 shows the correlation between the antioxidant activity and the cell viability.

**Figure 4:**
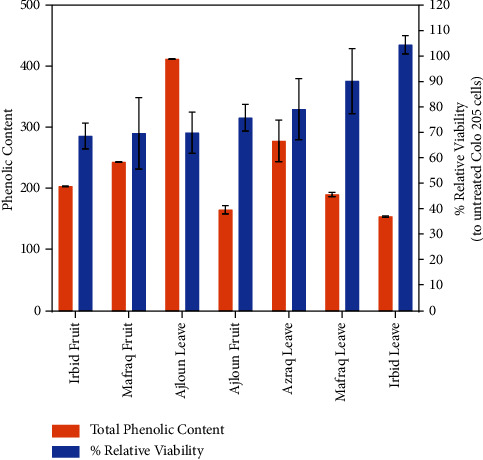
The correlation between the total phenolic content activity and the cell viability.

Additional correlation analyses were performed to assess the relationship between cell viability and total phenolic content in Figure 4. A notable association has been observed between the total phenolic content and cell viability. Among the fruits and leaves analyzed, Mafraq fruit and Azraq leaves exhibited the highest total phenolic content, concomitant with the lowest cell viability. No significant correlation was observed between the other samples, which could potentially be attributed to the presence of distinct phenolic compounds affecting the functionality of colon cancer cell lines. Further research is required to isolate all individual phenolic compounds and conduct biological assays to determine their effects.

## 4. Discussion

In this study, the primary focus was placed on the assessment of olive fruits and leaves, specifically from four different cultivars of *Olea europaea* L. in Jordan. It is essential to provide clarity on the rationale behind this specific focus and why other fruits or leaves were not included in the scope of our research.

The choice to concentrate on olive fruits and leaves was guided by several compelling reasons:

1. *Cultural and Regional Significance*. Olive cultivation has deep-rooted cultural and regional significance in the Mediterranean, including Jordan. Olive trees have been a fundamental part of the dietary and agricultural heritage of the region for millennia. As such, understanding the nutritional and health attributes of olive products, such as fruits and leaves, holds paramount importance for both local communities and the wider scientific community [25].
2. *Unique Bioactive Compounds*. Olive products, particularly olive leaves and fruits, are known for their distinctive bioactive compounds, including phenolic chemicals, flavonoids, and secoiridoids. These compounds have been extensively studied for their potential health benefits, making olive extracts a pertinent subject for research [26].
3. *Local Varietal Diversity*. Jordan hosts diverse olive cultivars that possess unique phenolic profiles and antioxidant capacities [11]. Investigating the variations within this local diversity provides valuable insights into the potential health benefits that can be attributed to different cultivars and geographical regions [27].
4. *Specific Health Focus*. The study was specifically designed to assess the potential health benefits of olive extracts for colon health. By concentrating on olive fruits and leaves, we were able to target a specific area of interest and examine the compounds that are most likely to be relevant to this health focus [11].

While our study centered on olive fruits and leaves, we acknowledge the rich diversity of fruits and leaves available for research. The decision to limit our focus was driven by the need for specificity and a well-defined research scope. However, we recognize the potential for future investigations to explore other fruits and leaves and their respective bioactive compounds, which may contribute to a more comprehensive understanding of the health benefits of various plant sources.

Our study's emphasis on olive fruits and leaves was guided by their cultural significance, unique bioactive compounds, local varietal diversity, and the specific health focus of colon health. We believe that our research contributes valuable insights into the potential health benefits of olive extracts, and we encourage further exploration of other plant sources in future studies to broaden our understanding of natural antioxidants and their impact on human health.

**Figure 5:**
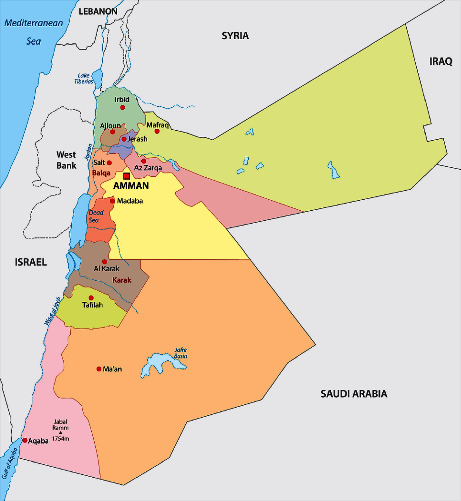
Geographical distribution of olive cultivars in Jordan (the figure was adapted from https://www.worldatlas.com/maps/jordan on 15/09/2023).

In this study, the results of the total phenolic content analysis showed that Ajloun leaves had the highest phenolic content among the samples, followed by Azraq leaves, Maraq fruit, Azraq fruit, Irbid fruit, Mafraq leaves, Ajloun fruit, and Irbid leaves. These findings are consistent with previous studies that have reported the presence of high levels of phenolic compounds in olive leaves and fruits [28]. Additionally, it was found that the fruits generally exhibited higher total phenolic content than the leaves, with the exception of Azraq and Ajloun leaves. This observation can likely be attributed to various environmental factors that influence the growth and development of plants, including the availability of nutrients, climate conditions, and soil composition [29]. While this study represents the first investigation into the variations in phenolic content and antioxidant properties among Jordanian leaves, it is essential to acknowledge the geographical and environmental diversity within Jordan. As indicated in Figure 5 on the Jordan map, Azraq, Ajloun, Irbid, and Mafraq are located in different regions with distinct climatic conditions and soil compositions. These geographical variations can indeed play a significant role in determining the phenolic content of leaves. Factors such as temperature, precipitation, soil type, and altitude can influence the production of secondary metabolites, including phenolic compounds, in plants [30]. Therefore, the differences in phenolic content observed among the regions may be a result of the unique environmental conditions each region offers for plant growth [31]. *Total Antioxidant Activity*. The antioxidant activity analysis revealed that the Irbid fruit extract had the highest antioxidant activity, while the Azraq fruit extract had the lowest. Furthermore, the results of the biological assay test conducted against colon cancer cells indicated that higher levels of antioxidant activity led to decreased cell viability, suggesting a correlation between apoptosis and antioxidant activity. These findings are consistent with previous studies that have reported the induction of apoptosis as a desired outcome in cancer treatment [32]. *Correlation Analyses*. The correlation analyses conducted in this study showed that there was a notable association between the total phenolic content and cell viability. Mafraq fruit and Azraq leaves exhibited the highest total phenolic content, which was concomitant with the lowest cell viability. However, no significant correlation was observed between the other samples. These results suggest that the presence of distinct phenolic compounds may affect the functionality of colon cancer cell lines, highlighting the need for further research to isolate individual phenolic compounds and determine their effects. Overall, the results of this study demonstrate the potential of olive extracts as anticancer agents, particularly in the induction of apoptosis. However, further studies are required to fully understand the mechanism of action and potential therapeutic applications of these extracts.

## 5. Future Study

Our future research will focus on elucidating the effects of phenolic compounds on human health, particularly in the context of their potential therapeutic applications. To achieve this, we will employ a multifaceted approach that combines several suitable techniques.

*Clinical Trials*. Given our objective to understand the practical implications of phenolic compounds, we will conduct controlled clinical trials involving human participants. These trials will provide direct insights into how phenolic compounds impact human health, encompassing aspects such as disease prevention, symptom alleviation, and overall well-being.

*Metabolomics*. To complement our clinical data, we will perform metabolomic analyses. This technique will allow us to examine changes in metabolic pathways and metabolite profiles in response to phenolic compound exposure. Mass spectrometry and NMR spectroscopy will be instrumental in identifying metabolic signatures associated with the compounds.

*Gene Expression Profiling*. To delve into the molecular mechanisms underlying the effects of phenolic compounds, we will conduct gene expression profiling. This technique will enable us to uncover changes in gene expression patterns in relevant tissues and cells, shedding light on the compounds' molecular modes of action.

*Cell Culture Studies*. In parallel, we will use cell culture studies to explore the cellular mechanisms affected by phenolic compounds. This approach will provide valuable insights into how these compounds influence cell behavior and health.

By combining these techniques, our research aims to provide a comprehensive understanding of the effects of phenolic compounds, from the molecular level to clinical outcomes. This approach will not only enhance our knowledge of the compounds' potential therapeutic benefits but also contribute to evidence-based healthcare strategies for improving human health and well-being.

## 6. Conclusion

The assessment of olive fruit and leaf extract chemical compositions in terms of antioxidant activity and total phenolic content had several attributes: (1) the ability to rank cultivated olive varieties for both fresh market and olive processing in terms of potential health benefits; many of the varieties studied were native to Jordan and had never been assessed; (2) encourage farmers to grow varieties which have higher total phenolic content and antioxidant activity that have added nutritional and health value. Finally, it is recommended to conduct a wider sampling in a future study of olive varieties over more than four regions to gain a much broader idea about the chemical composition of different olive varieties in Jordan.

## Figures and Tables

**Table 1:** The total phenolic content of the olive samples.

Sample name	Total phenol
Irbid fruit	205.0714286
Irbid leaves	155.3095238
Mafraq fruit	244.9047619
Mafraq leaves	191.5
Azraq fruit	239.3571429
Azraq leaves	279.3571429
Ajloun fruit	166.5
Ajloun leaves	413.6428571

**Table 2:** The antioxidant content of the olive samples.

Sample name	Antioxidant
Irbid fruit	473.0405431
Irbid leaves	304.4365121
Mafraq fruit	463.0417606
Mafraq leaves	332.800119
Azraq fruit	293.4918186
Azraq leaves	349.8816288
Ajloun fruit	425.5446781
Ajloun leaves	435.1060426

## Data Availability

The data will be available at the journal website after acceptance.
